# Environmental Justice and Pesticides in the Global South–A Scoping Review

**DOI:** 10.1007/s40572-026-00535-9

**Published:** 2026-04-16

**Authors:** Meryl Jagarnath, Leslie London, Iman Nuwayhid, Rima R. Habib

**Affiliations:** 1 Division of Environmental Health and Centre for Environmental and Occupational Health Research, School of Public Health, University of Cape Town, Anzio Rd., Observatory, 7925 South Africa; 2 Department of Environmental Health, Faculty of Health Sciences, American University of Beirut, Riad-El-Solh, P.O. Box 11-0236, Beirut, 1107 2020 Lebanon

**Keywords:** Toxic exposures, Pesticide policy, Environmental epidemiology, Structural inequality

## Abstract

**Purpose of Review:**

Pesticide exposure is an urgent environmental health issue in the Global South, where structural inequalities, weak regulation, and the export of banned chemicals from the Global North create disproportionate risks for marginalized populations. While environmental justice (EJ) frameworks are increasingly applied in environmental health research, their integration in pesticide research and interventions in the Global South has not been reviewed. This scoping review maps how EJ is conceptualized and operationalized in studies on pesticides in the Global South, identifying justice dimensions, methodological approaches, and interventions.

**Recent Findings:**

Fifty-four peer-reviewed and grey literature sources (1995 to 2024, English language) were reviewed using a five-dimensional EJ framework: distributive, procedural, recognition, capabilities-based, and epistemic justice. Distributive justice dominated, procedural and recognition justice were inconsistently addressed, and capabilities-based and epistemic justice were rarely applied. EJ applications varied regionally: Central and South American studies had a pluralistic approach to EJ. Sub-Saharan African (SSA) research emphasized acute exposure disparities but lacked epistemic justice. Southeast Asia focused on gender and caste-based inequities, and the Middle East and North African (MENA) region was notably under-represented, highlighting a significant geographic gap. Interventions included pesticide bans, litigation, advocacy, user training, and household adaptations, yet most reinforced individual responsibility rather than addressing structural drivers of pesticide use. Additional gaps include limited gender-specific analyses, scarce epidemiological evidence, and co-production of knowledge with affected communities.

**Summary:**

Pesticide-related EJ scholarship in the Global South remains uneven, with major regional, methodological, and epistemological gaps. Advancing EJ requires rights-based, community-led, gender-sensitive and epistemically inclusive approaches, strengthened regulations, and a global ban on prohibited pesticides.

**Supplementary Information:**

The online version contains supplementary material available at 10.1007/s40572-026-00535-9.

## Introduction

Pesticides are among the most widely used chemicals globally, with applications in agriculture, public health, and households [1]. In 2021, countries in the Global South accounted for more than half of global pesticide use [2], therefore a substantial proportion of the global population are exposed to associated health risks including neurotoxicity, endocrine disruption, reproductive harm, and cancer [3]. Exposure in these settings is driven not only by increased use of highly hazardous pesticides (HHPs) but also weak regulatory oversight and limited investment into alternatives to pesticide use [4, 5]. Unsafe pesticide handling, storage, application, and disposal practices are often cited as the main drivers of exposure in these settings [6, 7], but framing these risks as ‘misuse’ individualizes blame while obscuring the structural conditions that normalize the use of inherently toxic substances in marginalized settings, where agro-industrial production is prioritized over the health of workers, communities and the environment [8]. This reflects a broader environmental justice (EJ) concern, as the harms associated with pesticide exposure fall disproportionately on socially and economically marginalized groups with limited power to mitigate their risks. Additionally, the continued export of pesticides banned in high-income countries to lower-income countries [9]. further entrenches these inequities.

EJ, grounded in the equitable distribution of environmental risks and benefits related to pesticide use, has been extensively examined in the Global North, including pesticide drift and occupational exposures among racial and ethnic minorities [10, 11]. In contrast, pesticide-related EJ scholarship in the Global South is sparse [12], despite widespread exposure and distinct socio-political contexts shaped by colonial legacies, weaker regulatory protections and greater use of HHPs [5, 13]. This scarcity is compounded by underinvestment in environmental health research, limited capacity for exposure monitoring, dependence on externally defined research agendas and restricted access to sustained funding for longitudinal studies [5, 12, 14, 15]. These systemic barriers constrain the production of locally relevant evidence and reinforce the dominance of Global North perspectives in framing pesticide risk and EJ.

EJ has evolved to encompass distributive, procedural, recognition, capabilities-based, and epistemic dimensions [16]. Distributive justice addresses the unequal allocation of environmental goods and harms [17], such as the uneven burden of pesticide-related health risks. Procedural justice emphasizes fair and inclusive participation in environmental decision-making, ensuring that affected communities have a meaningful voice in shaping policies and regulations [18]. Recognitional justice focuses on the socio-cultural roots of oppression, including the historical and current marginalization of communities that are most exposed to toxicants yet remain invisible in environmental governance [18]. The capabilities approach draws attention to the foundational conditions required for individuals to lead flourishing lives, such as access to resources, institutions, and freedoms that support health and well-being [19]. More recently, epistemic justice has gained prominence as a critical lens, especially relevant in the context of environmental health issues in the Global South [20]. To date, only two reviews have explicitly addressed pesticides through an EJ perspective: (i) Isgren and Anderson [12] for sub-Saharan Africa (SSA), which primarily looked at distributive and procedural justice, and (ii) Donley et al. [21] for the United States, which examined structural racism and regulatory failures. Other regional reviews in Latin America and the Caribbean (LAC) [22, 23], Middle East and North Africa (MENA) [24–26], and SSA [13] have assessed health outcomes but not disparities or the structural determinants of exposure.

Recognizing these gaps, the aim of this scoping review is to map how pesticide-related environmental injustices are conceptualized, studied, and addressed in the Global South. To our knowledge, this is the first review to systematically assess whether and how pesticides are framed explicitly as an EJ issue in this context. Grounded in the multidimensional EJ framework [17], that integrates human rights perspectives [27], we identify patterns, interventions, gaps and future directions in the literature to advance research, advocacy, and policy. By centering the Global South, this review aims to advance more equitable global pesticide governance, aligning with Sustainable Development Goal (SDG) 10 on reducing inequality.

## Methods

We conducted a scoping review to identify whether and how EJ is conceptualized and operationalized in the academic and grey literature on pesticide exposure in the Global South, to synthesize: (i) the dimensions of EJ addressed (distributive, procedural, recognition, capabilities, and epistemic), and (ii) the regulatory, policy, and community-based interventions to address pesticide-related environmental injustices. A scoping review methodology is appropriate for this topic as it maps the volume and focus of the available evidence and can identify trends, gaps and future research directions [28]. We followed the methodological framework proposed by Arksey and Malley [29] and report the process according to the Preferred Reporting Items for Systematic Reviews and Meta-analyses (PRISMA) Extension for Scoping Reviews (PRISMA-ScR) [30]. This scoping review protocol is registered with the Open Science Framework (OSF) (DOI10.17605/OSF.IO/RBX2).

### Search Strategy

The identification of relevant studies was done by applying a search strategy developed with the assistance of a public health librarian from the Bongani Mayosi Health Sciences Library at the University of Cape Town. The full search strategy included specific Medical Subject Headings (MeSH) terms and keywords following the Population, Exposure, Comparison, Outcome, Study design (PECOS) framework recommended by the Joanna Briggs Institute (JBI) [31] (Supplemental material Table S1). In this case, population refers to populations in the Global South affected by pesticide use and governance, including smallholder farmers, agricultural workers, informal traders, rural and urban communities, men, women, children, and migrant labourers. Exposure relates to exposure to pesticides through occupational, environmental, and domestic pathways, including through handling, mixing, application, selling, drift, poisoning, and environmental contamination. Comparison in our study refers not to a controlled variable but an examination of how pesticide-related environmental injustices differ across socio-demographic groups, geographies, and governance contexts, as well as how varying conceptual frameworks and interventions influence outcomes, particularly between the Global North and Global South. Outcomes refer to manifestations of EJ (distributive, procedural, recognition, capabilities-based, epistemic), health and environmental impacts, and intervention outcomes (policy, advocacy, and research effects). Study design refers to primary research using qualitative, quantitative, or mixed methods designs. Grey literature from credible institutions that contain empirical data or case-based analysis. The search terms included keywords for pesticides combined with terms for EJ, combined with Global South countries using Boolean operators to ensure no crucial information was left out. We searched for pesticides including specific classes.

We took a wide definition of EJ including both justice and rights-based approaches [27] which is also in line with how EJ is conceptualized in the Global South as connected with wider social issues and greater awareness of patterns of exploitation [20]. Global South refers to countries in SSA, LAC, MENA, South East Asia and the Pacific who see themselves as a group [32] which are generally less prosperous than wealthy, industrialized nations such as the United States, Canada, Australia, New Zealand, Japan, and the member states of the European Union [32]. Specifically, we used the following regional groupings to describe the specific regions in the Global South: SSA includes 48 countries which are Angola, Benin, Botswana, Burkina Faso, Burundi, Cabo Verde, Cameroon, Central African Republic, Chad, Comoros, Congo (Democratic Republic), Congo (Republic of), Cote d’Ivoire, Equatorial Guinea, Eritrea, Eswatini (formerly Swaziland), Ethiopia, Gabon, Gambia (The), Ghana, Guinea, Guinea-Bissau, Kenya, Lesotho, Liberia, Madagascar, Malawi, Mali, Mauritania, Mauritius, Mozambique, Namibia, Niger, Nigeria, Rwanda, Sao Tome and Principe, Senegal, Seychelles, Sierra Leone, Somalia (Federal Republic of), South Africa, South Sudan, Tanzania, Togo, Uganda, Zambia, and Zimbabwe [33]. The LAC region is made up of 24 countries including Argentina, Bolivia, Brazil, Chile, Colombia, Costa Rica, Cuba, Dominican Republic, Ecuador, El Salvador, Guatemala, Guyana, Haiti, Honduras, Jamaica, Mexico, Nicaragua, Panama, Paraguay, Peru, Suriname, Trinidad and Tobago, Uruguay, and Venezuela [34]. The MENA region consists of 20 countries including Algeria, Bahrain, Djibouti, Egypt, Iran, Iraq, Jordan, Kuwait, Lebanon, Libya, Morocco, Oman, Qatar, Saudi Arabia, State of Palestine, Sudan, Syrian Arab Republic, Tunisia, United Arab Emirates, and Yemen [35]. The South East Asia and Pacific region is comprised of Afghanistan, Bangladesh, Bhutan, Brunei Darussalam, Cambodia, China, Cook Islands, Fiji, India, Indonesia, Kiribati, Korea DPR, Lao PDR, Malaysia, Maldives, Marshall Islands, Micronesia, Mongolia, Myanmar, Nauru, Nepal, Niue, Pakistan, Palau, Papua New Guinea, Philippines, Samoa, Solomon Islands, Sri Lanka, Thailand, Timor-Leste, Tokelau, Tonga, Tuvalu, Vanuatu, and Viet Nam [36]. Additional hedges and filters were employed to exclude non-English studies, studies conducted outside of the Global South, and animal studies.

Four electronic databases were searched on 10 February 2025 by MJ and LL, with assistance from a public health librarian from the Bongani Mayosi Health Sciences Library at the University of Cape Town. The databases searched were PubMed, Scopus, EBSCOhost (Africa-wide and the Biological and Agricultural Index), and Web of Science (Biological Index, CINAHL, and SciELO). We restricted articles to English-language sources; however, no limitation was placed on publication date. The complete search strings for the different databases and search results are included in Table S2 in supplemental material. The reference lists of all included articles were screened to capture any relevant publications missed by the search strategy.

Grey literature, including reports and dissertations, was searched during 24 to 28 March 2025. Google Scholar and PRIMO were searched using search terms “environmental justice” and “pesticides”. Additionally, the websites of relevant organizations were reviewed for reports, such as World Health Organization (WHO), United Nations Environment Programme (UNEP), Food and Agriculture Organization (FAO), International Labour Organization (ILO), United Nations Human Rights (UNHR) Special Rapporteur on Toxics and Human Rights, International Pollutants Elimination Network (IPEN), and Pesticides Action Network (PAN).

### Eligibility Criteria

The eligibility criteria were established considering that research on EJ and pesticides in the Global South is emerging, and there may be limited peer-reviewed studies. Therefore, we included both peer-reviewed journal articles and grey literature, such as reports from governments, non-government organizations, and international agencies. Studies were included if they met all the following criteria:

- The study type was primary research articles employing qualitative, quantitative, or mixed methods approaches, as well as grey literature such as reports from governments, international agencies, or non-governmental organizations. If a study was a review but included an embedded case study, it was included as a case study, and only the case study data were extracted.
- The study was published in English.
- The geographic scope of the study was any country in the Global South, including but not limited to regions in SSA, LAC, Asia the Pacific, and MENA.
- The study must examine pesticide use or exposure (occupational, environmental, or domestic) as a central research concern.
- The study must address EJ, either explicitly or implicitly, associated with pesticide exposure. Eligible studies included any engagement with justice dimensions: distributive, procedural, recognition, capabilities-based, or epistemic.

Studies were excluded if they met any one of the following criteria:

- The study was reviews, editorials, commentaries, opinions, preprints, or non-peer-reviewed sources (except for grey literature from reputable organizations).
- The study was not published in English.
- The study geographic scope was not focused on the Global South.
- Studies on pesticide use or impacts without any justice-related framing (e.g., studies reporting solely on exposure levels, toxicological findings, or ecological risk).
- Studies that focused exclusively on non-human subjects or institutionalized populations (e.g., prisons, psychiatric hospitals), unless pesticide exposure was addressed in a justice framework applicable to community or environmental settings.
- Studies that examined gender, occupational, or socioeconomic aspects of pesticide exposure but did not incorporate justice, equity, or rights-based lens were excluded.

### Study Selection

We used Endnote software [37] to import citations and remove duplicates. Subsequently, Rayyan reference management software [38] was used to perform a blinded two-stage screening process. A blinded, two-stage screening process was implemented using Rayyan software. Two independent reviewers, the first author (MJ) and a graduate assistant (ABR), screened the titles and abstracts in the first stage, followed by a full-text screening in the second stage (Supplemental Material Screening Form). In stage one (title and abstract screening), studies were retained if they (i) involved pesticide exposure in the Global South and (ii) mentioned or implied concepts related to EJ, inequity, or disparity. At this stage, we accepted studies that used justice-adjacent terms even if EJ was not explicitly stated, recognizing that authors may use varying terminology to describe EJ-related dimensions. Articles explicitly framed around EJ were identified during a subsequent round of full-text review. In stage two (full-text review), studies were assessed against refined criteria. In cases of discrepancies in study inclusion, both reviewers engaged in iterative discussions to resolve the conflicts and consulted a third reviewer (LL) to resolve disagreements.

### Data Charting

Two reviewers extracted relevant data using a pre-defined data charting sheet in Microsoft Excel and cross-checked each other’s entries to ensure consistency. For each included study, we extracted information on study characteristics, including geographical location (country or region), setting (rural or urban), framing (local, regional, or global), where local framing was focused on a specific community or context; regional framing addressed multiple countries and cross-border dynamics; and global framings linked pesticide issues to trade, governance, colonial legacies, or transnational corporate practices. We included year of publication, study duration (months or years), study design (e.g., ethnographic, qualitative, quantitative, mixed methods), data collection instruments (e.g., surveys, interviews, participant observations, health questionnaires, clinical blood tests, legal cases) and data analysis (e.g., narrative analysis, thematic analysis, descriptive statistics, logistic regression). We also extracted pesticide exposure details: study population (including sample size), demographic information (sex, age, occupation), pesticide class or compounds, source(s) (occupational, environmental, domestic), route(s) (dermal, inhalation, ingestion) and health outcome(s) (self-reported or clinical), EJ component (distributive, procedural, recognition, capabilities, epistemic), and interventions to mitigate environmental injustice (regulatory, legal, advocacy, household, individual).

To analyze how EJ was conceptualized and operationalized, we applied the five-dimensional analytical framework derived from the literature. Each study was coded for its explicit or implicit consideration of distributive, procedural, recognition, capabilities, and epistemic justice:

- Distributive justice was coded for studies that identified who is most affected, linking disparities to structural inequalities (e.g., by class, gender, race, geography, labour type), and/or connecting exposures to historical or global factors (e.g., colonial legacies, double standards, export of banned pesticides, global agrochemical markets).
- Procedural justice studies critiqued the exclusion of affected communities (e.g., farmers, informal workers, residents) from pesticide-related policymaking, regulation, or redress mechanisms, or inadequate enforcement of protections, violations of human rights, or studies calling for legal reform.
- Recognition justice studies noted stigmatization or invisibility of marginalized groups (e.g., illicit crop farmers, Indigenous people, informal vendors) or misrepresentation of their concerns.
- Capabilities-based justice studies showed how pesticide exposure undermines basic capabilities (e.g., health, bodily integrity, education, future prospects), linking harms to social determinants (e.g., poverty, malnutrition, lack of healthcare), or highlighting life-course impacts, especially for women and children.
- Epistemic justice studies critiqued exclusion of Indigenous or experiential knowledge, documented community-generated knowledge, or explored grassroots activism or alternative justice pathways.

Coding was inductive and iterative; initial readings informed the refinement of coding definitions, and studies were recoded as necessary through consensus-based discussion. Studies could be assigned to multiple EJ dimensions where applicable. Interventions were coded by type (e.g., community-led, policy-based, regulatory), scope (individual, community, structural), and stated objective.

### Data Analysis

Due to the heterogeneity in methods and outcomes, results were summarized in a narrative way. Where possible, the concentrations of pesticides and the confidence intervals of health outcomes were reported. For qualitative studies, a thematic synthesis of results was conducted based on Thomas and Harden [39]. This study is reported in accordance with PRISMA-ScR guidelines [30] (Supplemental material, Table S3).

## Results

**Fig. 1:**
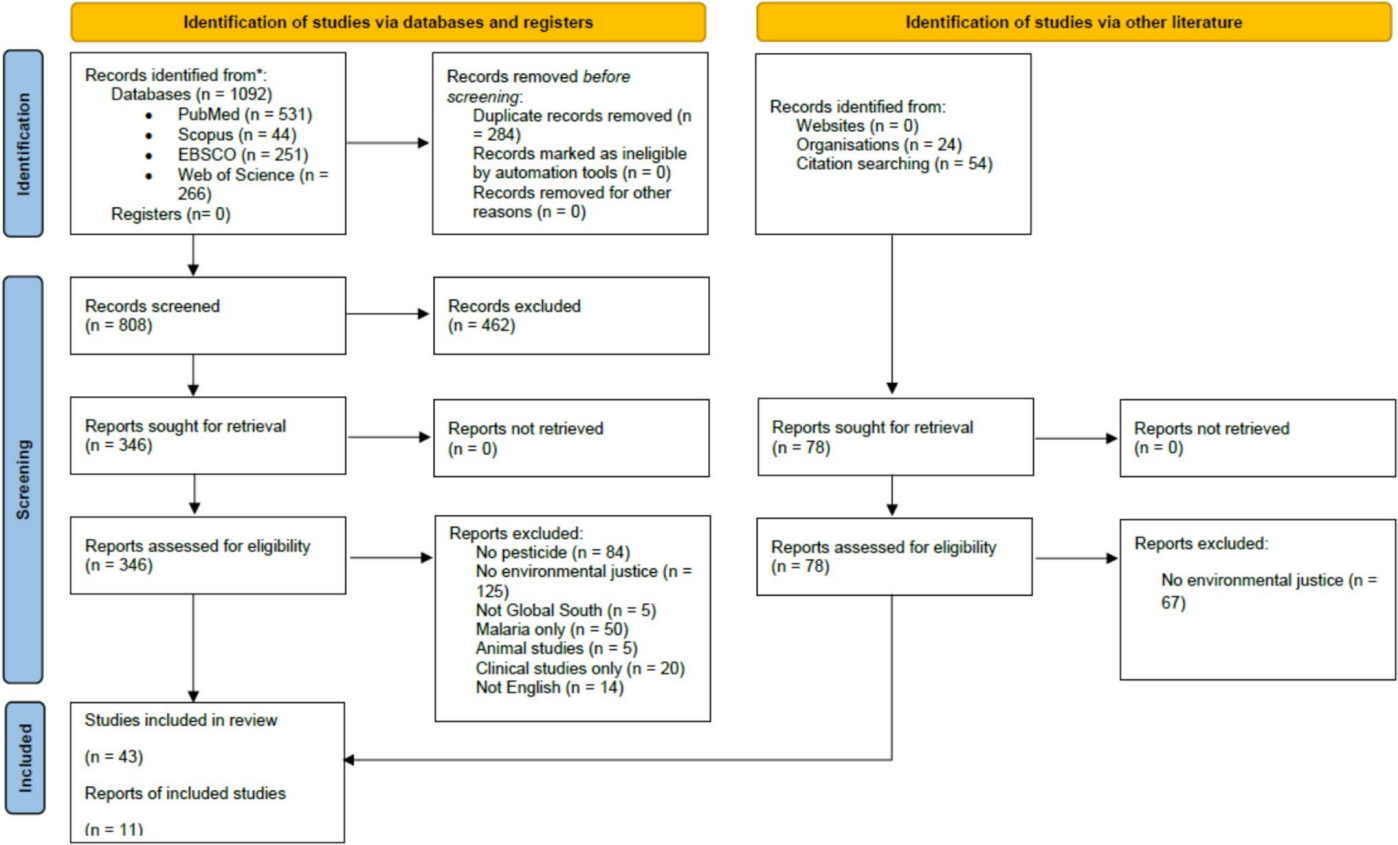
PRISMA-SCr flowchart

A total of 1092 publications were identified from searching databases, of which 284 articles were removed as duplicates. The remaining articles underwent title and abstract review, of which 462 did not meet the inclusion criteria and were excluded. The remaining 346 articles were assessed as full text reviews. Based on inclusion criteria, 303 publications were excluded because the studies (i) did not mention EJ or related terms (*n* = 125), (ii) did not mention pesticides or related terms (*n* = 84), (iii) were focused on malaria only (*n* = 50), (iv) were solely clinical studies (*n* = 20), (v) were non-English (*n* = 14), (vi) were not Global South (*n* = 5), or (vii) were animal studies only (*n* = 5). The database search was supplemented by grey literature searching of which 78 reports were identified and 11 included. Ultimately, we extracted data from 54 publications (Fig. 1) and also listed in Table S4 (List of included publications in Supplemental Materials).

### Descriptive Characteristics of Studies

**Fig. 2:**
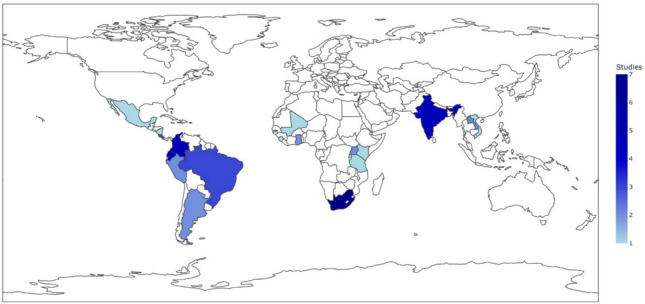
Map showing geographical distribution of included studies

The scoping review included 54 studies published between 1995 and 2024 comprising qualitative (*n* = 24), ethnographic (*n* = 9), quantitative (*n* = 6), and mixed methods (*n* = 4) studies, and reports (*n* = 11) (Table 1). The geographic distribution of the studies was concentrated in South America (*n* = 16) and SSA (*n* = 13), followed by Asia (*n* = 8), Central America (*n* = 6) and the MENA (*n* = 1) (Fig. 2). At the country level, studies in South Africa (*n* = 7), Colombia (*n* = 5), Ecuador (*n* = 3), and India (*n* = 5) were frequent. Most studies were in rural, agricultural settings (*n* = 37), with fewer in urban (*n* = 3) and peri-urban (*n* = 1) settings. More information on the characteristics of included studies is available in Table S5 in Supplemental Materials.

**Table 1:** Descriptive characteristics of included studies (*n* = 54)

Items	Number of studies *n* (%)
Region	
South America	16 (30)
SSA	13 (24)
Low- and Middle-Income Countries/Developing Countries as a single region	10 (18)
Asia	8 (15)
Central America	6 (11)
MENA	1 (2)
Publication Year	
≤ 2000	3 (6)
2001–2005	4 (7)
2006–2010	5 (9)
2011–2015	6 (11)
2016–2020	19 (35)
2021–2024	17 (31)
Study setting	
Rural	46 (85)
Urban and rural	4 (7)
Urban	3 (6)
Peri-urban	1 (2)
Study design	
Qualitative	24 (44)
Reports	11 (20)
Ethnographic	9 (17)
Quantitative	6 (11)
Mixed methods	4 (7)
Sources of pesticide exposure and pathway (some studies included more than one source and/or pathway)	
Occupational	36 (66)
• Applying pesticides	20 (37)
• Harvesting crops	5 (9)
• Disposal of empty pesticide containers	4 (7)
• Vendors in the informal pesticide market	4 (7)
• Storage of pesticide containers	3 (6)
Environmental exposures	27 (50)
• Spray drift into surrounding communities	15 (28)
• Take-home exposures	6 (11)
• Re-use of pesticide containers	4 (7)
• Pesticide-contaminated food/drink	2 (4)
Environmental justice dimensions (some studies included more than one dimension)	
Distributive justice	34 (63)
Procedural justice	30 (56)
Recognitional justice	8 (15)
Epistemic justice	7 (13)
Capabilities-based justice	2 (4)

### Sources of Pesticide Exposures

Occupational exposure was the predominant focus (*n* = 29), followed by combined occupational and environmental/community exposures (*n* = 16) and environmental-only exposures (*n* = 9) (Table 1). Common occupational pathways included direct contact during application, disposal, and handling contaminated clothing and equipment. Environmental exposures were primarily from pesticide drift, storage, and the informal sale of ‘street pesticides’ (agricultural chemicals or unregistered pesticides repackaged into unmarked containers) in urban areas, often linked to acute poisoning events among children.

### Manifestations of Environmental Injustices

The literature on environmental injustice issues related to pesticides in the Global South primarily engaged with distributive and procedural justice, with recognition, epistemic, and capabilities-justice less frequently addressed (Table 1). Distributive injustice reveals how pesticide harm disproportionately affects structurally vulnerable groups, such as smallholder farmers, plantation workers, indigenous peoples, migrant labourers, and the urban poor, due to intersecting social, economic and historical inequities. Procedural injustice highlights the exclusion of affected communities from decision-making and the inadequate enforcement of protective regulations. Recognition injustice focused on the marginalization and stigmatization of groups whose experiences and identities are often overlooked in pesticide policy. The capabilities approach connects pesticide exposure to broader socio-economic constraints, with the associated health impacts of pesticide exposure, limiting individuals’ opportunities for health and wellbeing. Epistemic injustice highlights the dominance of corporate and colonial scientific paradigms and the politicization of scientific uncertainty that marginalize local, indigenous and experiential knowledge; however epistemic justice also shows ways in which communities resist these injustices through counter-expertise and environmental stewardship.

### EJ in Epidemiology Studies

#### Overview of Studies

The reviewed literature comprised of six epidemiological studies that incorporated EJ, with the following study designs: retrospective observation [40], cross-sectional [41, 42], longitudinal [43], case–control [44] and ecological [57]. Table 2 presents the characteristics of these studies. Most studies were conducted in South American contexts–Colombia, Ecuador, and Brazil–followed by Central America and Africa. These studies focused on a range of pesticides including organophosphates, carbamates, and organochlorine compounds, used in rural agricultural settings. These studies investigated pesticide exposure not only as an occupational and environmental health hazard but also as a symptom of broader structural inequities. Several studies specifically targeted occupational groups within specific sectors such as floriculture [41, 44], smallholder farmers and their families [49], and vulnerable population such as child labourers [40], and illicit crop farmers [42]. Studies also investigated community-wide exposures surrounding agricultural fields [44], and population-level pesticide poisoning [45]. The outcomes measured ranged from biochemical indicators of pesticide absorption (e.g., acetylcholinesterase inhibition) to broader neurobehavioral and psychosocial health effects.

**Table 2:** Characteristics of epidemiological research on pesticide exposure and environmental (in)justice

Reference	Study location	Study region	Study setting	Study population, sample size and male-to-female ratio	Study design	Study duration	Data collection methods	Data analysis
Breilh et al. [41]	Granobles River Basin, Cayambe, Ecuador	South America	Rural	Agricultural workers aged between 18 to 69 years (*n* = 123) Male = 64.2% Female = 35.8%	Cross-sectional and clinical tests	2008	Questionnaires (Pentox and EpiStress) Clinical blood test for acetylcholinesterase (AChE)	Descriptive statistics Factor analysis Logistic Regression Canonical discriminant analysis
Cole et al. [43]	Central and Northen Ecuador	South America	Rural	Potato farm manager (*n* = 380): Male = 87% Female = 13% Household manager (*n* = 376): Male = 3% Female = 97%	Longitudinal	July 2005 to January 2007	Household surveys Neurological assessment	Descriptive statistics Multiple regression
Corriols and Aragorn [40]	Nicaragua	Central America	Rural and urban	Acute pesticide poisoning (APP) cases involving children aged 5–14 years engaged in agricultural work between 1995–2006 (*n* = 432) APP cases: Boys = 77% Girls = 23% Mortality (*n* = 6): Boys = 67% Girls = 33%	Retrospective observational	1995–2006	Poison surveillance data	Descriptive statistics
Creed et al. [44]	Naivasha and Mogotio, Kenya	Africa	Rural	Residents of Naivasha (industrial cut flower hub) and Mogotio (rural agricultural community) Naivasha socio-economic survey (*n* = 800) Male = 37% Female = 63% Mogotio socio-economic survey (*n* = 200): Male = 37% Females = 69% Biological samples (*n* = 89): Male = 34% Female = 66%	Case–control	-	Socio-economic survey Biological samples	Descriptive statistics Inferential statistics Random Forest Analysis
Moraes et al. [45]	Brazil	South America	Rural and urban	Pesticide poisoning cases from the national health database (*n* = 41254) Male = 56% Female = 44%	Ecological	2017 to 2019	Poisoning notifications	Descriptive statistics Correlation
Varona et al. [42]	Colombia	South America	Rural	Agricultural workers in an area growing illegal crops and have used pesticides in farming activities during the past two years (*n* = 112) Number of male and female participants not provided	Cross-sectional	2005 to 2006	Biological samples	Descriptive statistics

#### Vulnerable Populations and Exposure Disparities in Epidemiological Studies

In Ecuador, two studies investigated floriculture workers [41] and smallholder farming communities [43], which are populations characterized by low-income status, limited access to healthcare, and minimal institutional protections. Breilh et al. [41] emphasized the urgent need for low-cost, scalable tools to monitor chronic pesticide poisoning among floriculture workers, while Cole et al. [43] highlighted persistent neurobehavioral impairments in poorer communities despite pesticide safety training and education intervention programmes, due to the cumulative and lasting effects of socioeconomic disadvantage and the legacy of past pesticide exposures. Corriols and Aragorn [40] examined child labourers aged 5–14 years in Nicaragua, and found a high burden of acute pesticide poisoning (APP) among those working in agricultural sectors producing export crops, of which tobacco cultivation accounted for 46% of APP cases, while other export crops contributed a further 34% of APP cases. In Colombia, communities involved in illicit crop cultivation were studied, where residents were exposed to banned organochlorines under conditions of legal invisibility and neglect by public health systems [42]. In Brazil, Moraes et al. [45] focused on ethnicity and race, demonstrating higher poisoning-related morbidity and mortality among non-white populations due to associations with structural socioeconomic indicators. In Kenya, Creed et al. [44] identified both occupational and para-occupational exposures in and around the cut-flower industry, with a novel focus on gendered differences. Women living near both industrial flower and subsistence farming zones exhibited higher pesticide exposure and stress biomarkers, attributed to domestic pesticide use and caregiving responsibilities [44].

#### Metrics to Measure Inequality in Epidemiology

The studies employed a variety of metrics to operationalize inequality measurements in epidemiology. Corriols and Aragorn [40] measured underreporting of acute pesticide poisoning (APP) in child labourers, as a proxy for institutional neglect, revealing that 2% of an estimated 18,516 poisoning cases among child labourers were officially recorded. This stark discrepancy served as an index of systemic invisibility, compounded by the age and socioeconomic status of the affected population. Another study used occupational context and residential geography to capture disparities, noting significantly elevated concentrations of banned pesticides, such as Heptachlor, 4–4-DDE, and Aldrin in areas where illegal crops were cultivated, reflecting both legal marginalization and environmental vulnerability [42]. Cole et al. [43] developed an Unsatisfied Basic Needs Indicator (NBI) to capture multidimensional poverty and included housing quality, access to health and education, and employment status, in order to correlate persistent social deprivation with poorer cognitive outcomes and higher pesticide exposure. Another index was developed using employed relative risk calculations of pesticide poisoning by racial groups alongside the Human Development Index (HDI) to quantify racial disparities in pesticide-related poisonings in Brazil, pointing to systemic racial inequities in environmental health outcomes [45]. Spatial analysis between industrial (Naivasha) and non-industrial (Mogotio) areas, alongside gender-disaggregated data revealed pesticide exposure patterns and psychosocial stress burdens [44]. Women, identified as “sentinels” of exposure, had consistently higher acetylcholinesterase (AChE) inhibition and hair cortisol levels [44] highlighting the intersectionality of environmental and gender injustice.

## Discussion

This scoping review aimed to explore how EJ is conceptualized, studied, and operationalized in relation to pesticide exposure in the Global South. Our analysis of 54 studies shows that, in the Global South, EJ is often framed in terms of distributive justice. This emphasis on distributive justice highlights both the urgent need to identify inequities in pesticide exposure and a notable gap in addressing the full range of EJ dimensions [12, 17].

A critical limitation mentioned across the included studies is the absence of robust epidemiological data in the Global South to support EJ claims, which is confirmed by other studies noting the limited environmental health research and capacity for exposure monitoring in the Global South due to lack of research infrastructure and sustained funding and capacity for longitudinal studies [5, 12, 14, 15]. These systemic barriers constrain the production of locally relevant evidence and reinforce the dominance of Global North perspectives in framing pesticide risk and EJ [14, 15]. Health studies included in this review rely on self-reported symptoms [47], and clinical testing exists, however these tests are often limited by sample size, context, or access to diagnostics [42–44]. Studies claim that this evidence gap reinforces institutional skepticism and limits the capacity of affected communities to demonstrate health impacts of exposure [14]. Although epidemiological study designs frequently document disparities in pesticide exposure, they rarely challenge the social, political and epistemic factors that produce pesticide exposure disparities. In contrast, qualitative and ethnographic studies engage with recognition and epistemic justice [15, 45, 47–51]. This highlights how methodological silos shape the understanding (or lack thereof) of environmental injustice. Claims on environmental injustices emerge not solely from data gaps or regulatory failure, but from entrenched epistemic hierarchies, where certain types of knowledge are more valuable or credible than others, and the dominant conceptions of justice, of what is considered ‘fair’ or ‘just’ in society is shaped by whose needs and perspectives are considered legitimate when assessing environmental risks [14, 15, 20, 52]. For example, Donley et al. [21] observed that in the U.S. context, even strong epidemiological evidence can be sidelined when regulatory agencies are structurally aligned with agrochemical interests. Addressing pesticide-related environmental injustices in the Global South therefore requires not just more scientific evidence, but more inclusive and transformative ways of knowing that moves beyond cataloguing pesticide-related harms to actively including pluralistic EJ in pesticide research. Epidemiology needs to address this shift from documenting risk to co-producing knowledge with communities and including social and political factors in the contextualization of findings.

### Gaps in Conceptualizations of EJ

EJ is conceptualized and operationalized differently across regions of the Global South, shaped by historical, political and epistemological contexts unlike the Global North contexts where distributive justice is largely framed in terms of racial/ethnic or class/income exposure disparities [21, 53, 54]. In Latin America, EJ literature emphasizes the legacies of agro-industry, colonialism, extractive industries, and structural violence. EJ interventions are enacted through legal mobilizations, human rights claims, and epistemologies rooted in Indigenous and peasant knowledge systems. This reflects the pluralistic conceptualization of EJ claiming distributive, recognition, epistemic, and procedural justice approaches, informed by strong traditions of Latin American critical thought, social mobilization and solidarity [20, 52]. In Sub-Saharan Africa, the dominant EJ framing highlights acute exposures and weak regulatory enforcement in agricultural and informal urban settings. While the literature in Sub-Saharan Africa identifies exposure disparities, there is limited attention to social mobilizations or the development of regional EJ theories, as compared to Latin America. This agrees with Isgren and Andersson [12] who also noted the lack of mobilization on environmental injustices related to pesticide exposure for smallholder farmers in Sub-Saharan African, that may reflect deeper structural and epistemological factors. We found that the literature in sub-Saharan Africa emphasizes distributive justice, with less attention to recognition or epistemic dimensions. In South and Southeast Asia, the literature emphasizes caste- and class-based labour in plantations. EJ is often framed through a capabilities lens—examining how pesticide harms constrain life chances, especially for women and children [46, 51, 55], however epistemic justice remains underdeveloped relative to Latin America [14].

The MENA region is underrepresented, with only one study included. This gap reflects the observation by Braverman [54] that EJ scholarship is scarce in the MENA region, particularly in occupied territories. This absence is concerning given the region’s complex socio-political context and significant pesticide-related health [41]. Several pesticide-related studies from the region on human health [56–60] and the environment [61, 62] document exposure patterns, health risks and regulatory shortcomings, but do not conceptualize these issues within an EJ framework. Nevertheless, the absence of explicit EJ framings in MENA pesticide research should not be misinterpreted as the absence of environmental injustices. For instance, in the occupied Palestinian territories, pesticides are often purchased in Israel and distributed to Palestinian farmers [57]. This practice creates dependency on external agro-industrial inputs and limits local control over food production. In response to this practice, agro-activism in Palestine has prioritized heirloom seed sharing and the use of organic inputs to reduce reliance on foreign aid and Israeli products in order to reclaim their food systems and reduce the health and environmental risks associated with chemical-intensive agriculture [63]. Another significant environmental injustice issue is the aerial spraying of glyphosate by Israeli occupying forces in the Gaza Strip since 2014 [64, 65], a practice that parallels the experiences of Colombian farmers exposed to glyphosate spraying by the US military [66] and recalls historical instances of using herbicides in war such as the use of Agent Orange in Vietnam [67]. This spraying has destroyed arable land, undermining the livelihoods of Gazan farmers and limiting community access to food-producing resources [65]. However, these practices are often framed as humanitarian crises [65]. This framing emphasizes immediate response and protection under International Humanitarian Law, which according to Qandeel [68] is insufficient for safeguarding the environment under conditions of military occupation, thus deflecting from the issue of environmental injustice.

From an EJ perspective, pesticide use, control over agricultural inputs and land degradation are outcomes of structural inequities. In the occupied Palestinian territories, these harms are produced by Israeli settler colonial strategies, in which the settler society seeks to replace the Indigenous population, and then represent itself as the new native population to legitimize their territorial control [54]. The Palestinian case therefore highlights how environmental harms cannot be disentangled from geopolitical and colonial contexts, and why frameworks developed in one region of the Global South may not be appropriate for another. This underscores the need for a contextualized and pluralistic approach to EJ and also challenges the tendency to treat the Global South as a homogenous entity.

Most included studies used qualitative or ethnographic methods that described lived experiences of injustice, yet quantitative and clinical epidemiological work was limited and often did not engage explicitly with EJ concepts. We also did not identify studies co-authored with community-based organisations or directly affected populations, revealing an important procedural and epistemic justice gap. This absence reflects systemic barriers to publishing community-engaged research and limits the incorporation of lived experience in defining research questions and interpretations. Despite these limitations, this review provides the first systematic synthesis of how EJ is conceptualised and operationalised in pesticide-related research across the Global South. It offers an empirical base for future scholarship, policy development and advocacy by highlighting where research is concentrated and where gaps remain. Ultimately, addressing these geographic, methodological and epistemological gaps will be critical for advancing more inclusive and justice-centred environmental health research in structurally marginalised populations in the Global South and North.

### Recommendations for EJ and Pesticide Research and Advocacy in the Global South

To strengthen pesticide governance and contribute to more equitable environmental health globally, aligned with Sustainable Development Goal 10 on reducing inequality, we recommend that future research, advocacy, and policy efforts:

- Strengthen EJ and pesticide research foundations:
  ○ Adopt pluralistic frameworks that incorporate recognition, procedural, epistemic, and capabilities-based justice alongside distributive justice concerns.
  ○ Integrate gender-sensitive approaches, ensuring parity in representation of sex and gender to analyse differential exposures and vulnerabilities.
  ○ Expand research on urban and underexplored exposure pathways, including domestic use and vector control.
  ○ Prioritize context-specific research in under-represented regions, specifically the MENA region. Given the scarcity of scholarship explicitly linking pesticide exposure to EJ in MENA, researchers in environmental epidemiology and exposure science should be encouraged to integrate EJ conceptual frameworks into their study design and policy recommendations, including situating pesticide exposures within the regions context, recognizing how land governance, agricultural inputs and socio-political conflicts, and military occupation influence pesticide risks and inequalities.
- Deepen community-engaged and contextual research:
  ○ Engage directly with communities, through participatory and co-produced research that centres lived experience and local epistemologies.
  ○ Support the development of contextualized EJ frameworks grounded in regional histories, power dynamics, and knowledge systems.
  ○ Foster interdisciplinary and transdisciplinary collaborations linking health, human rights, and community organising.
- Advance advocacy and policy change:
  ○ Address the weaponization of uncertainty and advocate for policy responses aligned with the precautionary principle.
  ○ Advocate for a global ban on the export of pesticides that are prohibited for use in their countries of production, to prevent double standards and reduce transboundary environmental injustice.
- Foster interdisciplinary and transdisciplinary collaborations that link environmental health, human rights, and community organizing.
- Support the development of contextualized EJ frameworks that are grounded in regional histories, power dynamics and knowledge systems.

### Limits and Strengths of the Review

This scoping review has several limitations that should be considered. First, it was restricted to English-language publications due to the authors’ linguistic capacities and database access. This likely excluded important studies in Spanish, Portuguese, Arabic and French, underrepresenting pesticide-intensive regions in the Global South. Although we included peer-reviewed and some grey literature, our grey literature search focused on reports from internationally recognized organisations, which may have overlooked locally produced or activist scholarship. Geographical gaps were evident, with one study from MENA, and parts of SSA, Southeast Asia and Pacific not represented, reflecting broader structural inequalities in academic publishing [69], but also but also in the difficulty of delineating regions consistently; for example, deciding where Asia ends and the Middle East begins, or which North African countries are included. These ambiguities can complicate comparisons across studies and potentially influence regional analyses and conclusions. While literature on pesticide use and exposure are available in the MENA region, they rarely employ explicit EJ framing, resulting in their exclusion from our synthesis under the search string and/or the define inclusion criteria. This absence is an important finding, underscoring the need for pesticide research in the region to integrate EJ. Most included studies used qualitative or ethnographic methods that described lived experiences of injustice, yet quantitative and clinical epidemiological work was limited and often did not engage explicitly EJ concepts. We also did not identify studies co-authored with community-based organisations or directly affected populations, revealing an important procedural and epistemic justice gap. This absence reflects systemic barriers to publishing community-engaged research and limits the incorporation of lived experience in defining research questions and interpretations. Despite these limitations, this review provides the first systematic synthesis of how EJ is conceptualised and operationalised in pesticide-related research across the Global South. It offers an empirical base for future scholarship, policy development and advocacy by highlighting where research is concentrated and where gaps remain. Ultimately, addressing these geographic, methodological and epistemological gaps will be critical for advancing more inclusive and justice-centred environmental health research in structurally marginalised populations in the Global South and North.

## Conclusion and Future Directions

This scoping review provides a systematic synthesis of how EJ is conceptualized, operationalized, studied and enacted in relation to pesticide exposure in the Global South. Our findings highlight a growing but uneven integration of justice frameworks, the dominance of distributive justice, and less application of capabilities-based and epistemic justice. Geographical gaps are evident, with minimal contribution from the MENA region, where pesticide use is well-documented but rarely examined through an explicitly EJ lens. Future research should also prioritise under-represented contexts, including urban environments and non-agricultural exposure pathways. Achieving EJ for pesticide-exposed populations in the Global South and North requires moving beyond individualized risk framings toward rights-based, community-driven, and transformative approaches. Only by confronting the structural, epistemic and institutional foundations of environmental harm can research meaningfully contribute to the realization of dignity, health and justice for communities most impacted by pesticide use.

## Key References

- Arancibia F, Motta R. Undone Science and Counter-Expertise: Fighting for Justice in an Argentine Community Contaminated by Pesticides. Science As Culture. 2019;28(3): 277—302.
  - ○ This article examines how communities in Argentina exposed to aerial spraying of glyphosate produced their own knowledge to contest state and industry denial, demonstrating grassroots struggles for environmental justice.
- Donley N, Bullard DR, Economos J, Figueroa I, Lee J, Liebman KA, Martinez ND, Shafiei F. Pesticides and environmental injustice in the USA: root causes, current regulatory reinforcement and a path forward. BMC Public Health. 2022;22(1).
  - ○ This paper analyses how pesticide use in the United States perpetuates environmental injustice, tracing historical and regulatory factors that disproportionately burden marginalized communities.
- Isgren E, Andersson E. An Environmental Justice Perspective on Smallholder Pesticide Use in Sub-Saharan Africa. Journal Of Environment & Development. 2021;30(1):68—97.
  - ○ This study offers an environmental justice lens on pesticide use among smallholder farmers in Sub-Saharan Africa, highlighting unequal risks and barriers to safer alternatives.
- Sony RK, Münster D, Krishnan S. What counts as evidence? Examining the controversy over pesticide exposure and etiology in an environmental justice movement in Kerala, India. Environmental Sociology. 2023;9(2):148—164.
  - ○ This article analyses the contested politics of evidence in India, demonstrating how communities challenge official narratives that dismiss links between pesticide exposure and health outcomes.
- Utyasheva L, Rother H-A, London L, Eddleston M. Stop blaming the farmer: Dispelling the myths of ‘misuse’ and ‘safe’ use of pesticides to protect health and human rights. Journal of Human Rights. 2024. 23(3):231—252.
  - ○ This article critiques the dominant narrative of blaming users for misuse and calls for a rights-based approach to address the structural drivers of pesticide harm.

## Supplementary Information

Below is the link to the electronic supplementary material.

Supplementary file1 (DOCX 87 KB)

## Data Availability

No datasets were generated or analysed during the current study.
