# Supplementary material for: Environmental Justice and Pesticides in the Global South–A Scoping Review
Source: Curr Environ Health Rep. 2026 Apr 16;13(1):17. doi: 10.1007/s40572-026-00535-9 (PMC13086650; doi:10.1007/s40572-026-00535-9)
Supplement: Supplementary file 1 — Supplementary file1 (DOCX 87 KB) [file 40572_2026_535_MOESM1_ESM.docx]

**Supplemental Materials**

**Title:**

Environmental Justice and Pesticides in the Global South- A Scoping Review

**Authors**

Meryl Jagarnath^1^, Leslie London ^1^, Iman Nuwayhid ^2^, Rima R. Habib ^2^

Affiliations:

^1^Division of Environmental Health and Centre for Environmental and Occupational Health, School of Public Health and Family Medicine, University of Cape Town, Anzio Rd., Observatory 7925, South Africa.

^2^Department of Environmental Health, Faculty of Health Sciences, American University of Beirut, P.O. Box 11-0236, Riad-El-Solh, 1107 2020, Beirut, Lebanon.

Contents

[Table S1. PECOS Statement and inclusion/exclusion criteria 2](#_Toc202872287)

[Table S2. Search strategies employed in the scoping review 3](#_Toc202872288)

[Screening Form 9](#_Toc202872289)

[Table S3. Preferred Reporting Items for Systematic reviews and Meta-Analyses extension for Scoping Reviews (PRISMA-ScR) Checklist 11](#_Toc202872290)

[Table S4. List of Included Studies 13](#_Toc202872291)

[Table S5: Characteristics of included studies 18](#_Toc202872292)

# Table S1. PECOS Statement and inclusion/exclusion criteria

| Study aspect | Inclusion criteria | Exclusion criteria |
| --- | --- | --- |
| Population | Populations in the Global South affected by pesticide use and governance, including but not limited to smallholder farmers, agricultural workers, informal traders, rural and urban communities, men, women, children and migrant labourers. | Studies not conducted in Global South countries or studies on non-human populations. |
| Exposure | Pesticide exposure via environmental, occupational, or domestic pathways, including handling, mixing, application, selling, drift, poisoning, reuse of containers, or environmental contamination. Also includes studies on specific pesticide compounds or classes. | Studies that do not involve pesticide exposure or studies that examine ecological or toxicological effects without reference to human exposure or justice concerns. |
| Comparator | No comparison group is required. Studies may examine differential pesticide-related harms across socio-demographic groups (e.g. gender, race, class), geographies (e.g. urban vs rural), or between Global North and Global South contexts. | Studies lacking disparities framing were excluded. |
| Outcomes | Studies must examine at least one of the following: (i) justice dimensions (distributive, procedural, recognition, capabilities, or epistemic); (ii) health or environmental impacts linked to justice or human rights concerns; or (iii) outcomes of interventions (legal, policy, community-level, advocacy) aimed at addressing pesticide-related injustices. | Studies that do not engage with environmental justice, equity, or rights-based dimensions; studies on health or exposure outcomes without addressing justice or disparity; studies in gender, socioeconomic status, or occupation without an explicit justice lens. |
| Study design | Primary research using quantitative, qualitative, or mixed-methods designs. Also include grey literature from global, national and local institutions (e,g. WHO, UNEP, FAO, IPEN, national governments, civil society organizations) that contain empirical data or case study analysis. | Systematic reviews, scoping reviews, narrative reviews, opinion pieces, commentaries, and preprints. |
| Language | Published in English. | Not published in English |

# Table S2. Search strategies employed in the scoping review

| Databases | Strategy | Results |
| --- | --- | --- |
| PubMed | Search: (((("Environmental Justice"[Mesh]) OR ("Environmental justice" OR "environmental injustice" OR discrimination OR "environmental equity" OR "environmental inequity" OR "environmental inequality" OR "environmental racism" OR "health disparity" OR "environmental disparity" OR inequity OR inequality OR "human rights" OR "environmental equities" OR "environmental inequities" OR "environmental rights" OR "environmental inequalities" OR "health disparities" OR "human right" OR "environmental disparities")) AND (("Pesticides"[Mesh]) OR (Pesticide[Text Word] OR "plant protection product"[Text Word] OR PPP[Text Word] OR herbicide[Text Word] OR fungicide[Text Word] OR insecticide[Text Word] OR acaricide[Text Word] OR nematicide[Text Word] OR molluscicide[Text Word] OR rodenticide[Text Word] OR biocide[Text Word] OR bactericide[Text Word] OR "herbal pesticide"[Text Word] OR biopesticide[Text Word] OR nanopesticide[Text Word] OR biopesticide[Text Word] OR agrichemical[Text Word] OR agrochemical[Text Word] OR pest[Text Word] OR "pest control"[Text Word] OR "plant protection products"[Text Word]))) AND (Global south OR Afghanistan OR Algeria OR Angola OR Antigua and Barbuda OR Argentina OR Aruba OR Azerbaijan OR Bahamas OR Bahrain OR Bangladesh OR Barbados OR Belize OR Benin OR Bhutan OR Bolivia OR Botswana OR Brazil OR Brunei OR Burkina Faso OR Burundi OR Cabo Verde OR Cambodia OR Cameroon OR Central African Republic OR Chad OR Chile OR China OR Colombia OR Comoros OR Congo OR Costa Rica OR Côte d'Ivoire OR Cuba OR Djibouti OR Dominica OR Dominican Republic OR DRC OR Ecuador OR Egypt OR El Salvador OR Equatorial Guinea OR Eritrea OR Eswatini OR Ethiopia OR Fiji OR Gabon OR Gambia OR Ghana OR Grenada OR Guadeloupe OR Guatemala OR Guinea OR Guinea-Bissau OR Guyana OR Haiti OR Honduras OR India OR Indonesia OR Iran OR Iraq OR Jamaica OR Jordan OR Kazakhstan OR Kenya OR Kiribati OR Kuwait OR Kyrgyzstan OR Laos OR Lebanon OR Lesotho OR Liberia OR Libya OR Madagascar OR Malawi OR Malaysia OR Maldives OR Mali OR Mauritania OR Mauritius OR Mexico OR Micronesia OR Mongolia OR Morocco OR Mozambique OR Myanmar OR Namibia OR Nepal OR Nicaragua OR Niger OR Nigeria OR Oman OR Pakistan OR Palau OR Palestine OR Panama OR Papua New Guinea OR Paraguay OR Peru OR Philippines OR Qatar OR Rwanda OR Samoa OR Sao Tome and Principe OR Saudi Arabia OR Senegal OR Seychelles OR Sierra Leone OR Solomon Islands OR Somalia OR South Africa OR South Sudan OR Sri Lanka OR "St Vincent and Grenadines" OR Sudan OR Suriname OR Syria OR Tajikistan OR Tanzania OR Thailand OR Timor OR Togo OR Tonga OR Trinidad and Tobago OR Tunisia OR Turkey OR Turkmenistan OR Tuvalu OR Uganda OR United Arab Emirates OR Uruguay OR Uzbekistan OR Vanuatu OR Venezuela OR Vietnam OR Yemen OR Zambia OR Zimbabwe OR Swaziland)) NOT (insects) | 531 |
| EBSCO | #1  ("Environmental justice" OR "environmental injustice" OR "environmental equity" OR "environmental inequity" OR "environmental inequality" OR "environmental racism" OR "health disparity" OR "environmental disparity" OR inequit* OR inequalit* OR "human right*" OR "environmental equities" OR "environmental inequities" OR "environmental rights" OR "environmental inequalities" OR "health disparities" OR "environmental disparities" )  #2  (Pesticide* OR "plant protection product" OR PPP OR herbicide* OR fungicide* OR insecticide* OR acaricide* OR nematicide* OR molluscicide* OR rodenticide* OR biocide* OR bactericide* OR "herbal pesticide*" OR biopesticide* OR nanopesticide* OR agrichemical* OR agrochemical* OR pest* OR "pest control" OR "plant protection products")  #3  Global south OR Afghanistan OR Algeria OR Angola OR Antigua AND Barbuda OR Argentina OR Aruba OR Azerbaijan OR Bahamas OR Bahrain OR Bangladesh OR Barbados OR Belize OR Benin OR Bhutan OR Bolivia OR Botswana OR Brazil OR Brunei OR Burkina Faso OR Burundi OR Cabo Verde OR Cambodia OR Cameroon OR Central African Republic OR Chad OR Chile OR China OR Colombia OR Comoros OR Congo OR Costa Rica OR Côte d'Ivoire OR Cuba OR Djibouti OR Dominica OR Dominican Republic OR DRC OR Ecuador OR Egypt OR El Salvador OR Equatorial Guinea OR Eritrea OR Eswatini OR Ethiopia OR Fiji OR Gabon OR Gambia OR Ghana OR Grenada OR Guadeloupe OR Guatemala OR Guinea OR Guinea-Bissau OR Guyana OR Haiti OR Honduras OR India OR Indonesia OR Iran OR Iraq OR Jamaica OR Jordan OR Kazakhstan OR Kenya OR Kiribati OR Kuwait OR Kyrgyzstan OR Laos OR Lebanon OR Lesotho OR Liberia OR Libya OR Madagascar OR Malawi OR Malaysia OR Maldives OR Mali OR Mauritania OR Mauritius OR Mexico OR Micronesia OR Mongolia OR Morocco OR Mozambique OR Myanmar OR Namibia OR Nepal OR Nicaragua OR Niger OR Nigeria OR Oman OR Pakistan OR Palau OR Palestine OR Panama OR Papua New Guinea OR Paraguay OR Peru OR Philippines OR Qatar OR Rwanda OR Samoa OR Sao Tome AND Principe OR Saudi Arabia OR Senegal OR Seychelles OR Sierra Leone OR Solomon Islands OR Somalia OR South Africa OR South Sudan OR Sri Lanka OR "St Vincent and Grenadines" OR Sudan OR Suriname OR Syria OR Tajikistan OR Tanzania OR Thailand OR Timor OR Togo OR Tonga OR Trinidad AND Tobago OR Tunisia OR Turkey OR Turkmenistan OR Tuvalu OR Uganda OR United Arab Emirates OR Uruguay OR Uzbekistan OR Vanuatu OR Venezuela OR Vietnam OR Yemen OR Zambia OR Zimbabwe OR Swaziland  #4  insect OR insects  #5  #1 AND #2 AND #3  #6  #5 NOT #4 | 251 |
| Web of Science | #1  ( "Environmental justice" OR "environmental injustice" OR "environmental equity" OR "environmental inequity" OR "environmental inequality" OR "environmental racism" OR "health disparity" OR "environmental disparity" OR inequit* OR inequalit* OR "human right*" OR "environmental equities" OR "environmental inequities" OR "environmental rights" OR "environmental inequalities" OR "health disparities" OR "environmental disparities" ) (Topic) and Preprint Citation Index (Exclude – Database)  #2  ( pesticide* OR "plant protection product" OR ppp OR herbicide* OR fungicide* OR insecticide* OR acaricide* OR nematicide* OR molluscicide* OR rodenticide* OR biocide* OR bactericide* OR "herbal pesticide*" OR biopesticide* OR nanopesticide* OR agrichemical* OR agrochemical* OR pest* OR "pest control" OR "plant protection products" ) (Topic) and Preprint Citation Index (Exclude – Database)  #3  ( "Global south" OR afghanistan OR algeria OR angola OR "Antigua and Barbuda" OR argentina OR aruba OR azerbaijan OR bahamas OR bahrain OR bangladesh OR barbados OR belize OR benin OR bhutan OR bolivia OR botswana OR brazil OR brunei OR "Burkina Faso" OR burundi OR "Cabo Verde" OR cambodia OR cameroon OR "Central African Republic" OR chad OR chile OR china OR colombia OR comoros OR congo OR costa AND rica OR "Côte d'Ivoire" OR cuba OR djibouti OR dominica OR "Dominican Republic" OR drc OR ecuador OR egypt OR "El Salvador" OR "Equatorial Guinea" OR eritrea OR eswatini OR ethiopia OR fiji OR gabon OR gambia OR ghana OR grenada OR guadeloupe OR guatemala OR guinea OR guinea-bissau OR guyana OR haiti OR honduras OR india OR indonesia OR iran OR iraq OR jamaica OR jordan OR kazakhstan OR kenya OR kiribati OR kuwait OR kyrgyzstan OR laos OR lebanon OR lesotho OR liberia OR libya OR madagascar OR malawi OR malaysia OR maldives OR mali OR mauritania OR mauritius OR mexico OR micronesia OR mongolia OR morocco OR mozambique OR myanmar OR namibia OR nepal OR nicaragua OR niger OR nigeria OR oman OR pakistan OR palau OR palestine OR panama OR "Papua New Guinea" OR paraguay OR peru OR philippines OR qatar OR rwanda OR samoa OR "Sao Tome and Principe" OR "Saudi Arabia" OR senegal OR seychelles OR "Sierra Leone" OR "Solomon Islands" OR somalia OR "South Africa" OR "South Sudan" OR "Sri Lanka" OR "St Vincent and Grenadines" OR sudan OR suriname OR syria OR tajikistan OR tanzania OR thailand OR timor OR togo OR tonga OR "Trinidad and Tobago" OR tunisia OR turkey OR turkmenistan OR tuvalu OR uganda OR "United Arab Emirates" OR uruguay OR uzbekistan OR vanuatu OR venezuela OR vietnam OR yemen OR zambia OR zimbabwe OR swaziland ) (Topic) and Preprint Citation Index (Exclude – Database)  #4  (insect OR insects) (Topic) and Preprint Citation Index (Exclude – Database)  #5  #1 AND #2 AND #3 and Preprint Citation Index (Exclude – Database)  #6  #5 NOT #4 and Preprint Citation Index (Exclude – Database)  #7  #5 NOT #4 and Preprint Citation Index (Exclude – Database) and Biological Abstracts or SciELO Citation Index (Database) | 266 |
| Scopus | ( ( TITLE-ABS-KEY ( ( "Environmental justice" OR "environmental injustice" OR "environmental equity" OR "environmental inequity" OR "environmental inequality" OR "environmental racism" OR "health disparity" OR "environmental disparity" OR inequit* OR inequalit* OR "human right*" OR "environmental equities" OR "environmental inequities" OR "environmental rights" OR "environmental inequalities" OR "health disparities" OR "environmental disparities" ) ) ) AND ( TITLE-ABS-KEY ( ( pesticide* OR "plant protection product" OR ppp OR herbicide* OR fungicide* OR insecticide* OR acaricide* OR nematicide* OR molluscicide* OR rodenticide* OR biocide* OR bactericide* OR "herbal pesticide*" OR biopesticide* OR nanopesticide* OR agrichemical* OR agrochemical* OR pest* OR "pest control" OR "plant protection products" ) ) ) AND ( TITLE-ABS-KEY ( ( "Global south" OR afghanistan OR algeria OR angola OR "Antigua and Barbuda" OR argentina OR aruba OR azerbaijan OR bahamas OR bahrain OR bangladesh OR barbados OR belize OR benin OR bhutan OR bolivia OR botswana OR brazil OR brunei OR "Burkina Faso" OR burundi OR "Cabo Verde" OR cambodia OR cameroon OR "Central African Republic" OR chad OR chile OR china OR colombia OR comoros OR congo OR costa AND rica OR "Côte d'Ivoire" OR cuba OR djibouti OR dominica OR "Dominican Republic" OR drc OR ecuador OR egypt OR "El Salvador" OR "Equatorial Guinea" OR eritrea OR eswatini OR ethiopia OR fiji OR gabon OR gambia OR ghana OR grenada OR guadeloupe OR guatemala OR guinea OR guinea-bissau OR guyana OR haiti OR honduras OR india OR indonesia OR iran OR iraq OR jamaica OR jordan OR kazakhstan OR kenya OR kiribati OR kuwait OR kyrgyzstan OR laos OR lebanon OR lesotho OR liberia OR libya OR madagascar OR malawi OR malaysia OR maldives OR mali OR mauritania OR mauritius OR mexico OR micronesia OR mongolia OR morocco OR mozambique OR myanmar OR namibia OR nepal OR nicaragua OR niger OR nigeria OR oman OR pakistan OR palau OR palestine OR panama OR "Papua New Guinea" OR paraguay OR peru OR philippines OR qatar OR rwanda OR samoa OR "Sao Tome and Principe" OR "Saudi Arabia" OR senegal OR seychelles OR "Sierra Leone" OR "Solomon Islands" OR somalia OR "South Africa" OR "South Sudan" OR "Sri Lanka" OR "St Vincent and Grenadines" OR sudan OR suriname OR syria OR tajikistan OR tanzania OR thailand OR timor OR togo OR tonga OR "Trinidad and Tobago" OR tunisia OR turkey OR turkmenistan OR tuvalu OR uganda OR "United Arab Emirates" OR uruguay OR uzbekistan OR vanuatu OR venezuela OR vietnam OR yemen OR zambia OR zimbabwe OR swaziland ) ) ) ) AND NOT ( TITLE-ABS-KEY ( ( insect OR insects ) ) ) | 44 |
| Total results | | 1091 |

# Screening Form

**Stage 1 Title and Abstract Screening Form**

Instructions: Answer the questions in order. If the answer is NO, exclude the study and record the reason in Excel. If YES, proceed to the next question.

1. Is the publication in English?

- Yes **→** go to Q2
- No **→** exclude and record reason

1. Does the publication focus on a country or region in the Global South?

- Yes or maybe **→** go to Q3
- No **→** exclude and record reason

1. Does the publication report on pesticide exposure (occupational, environmental, community, domestic)?

- Yes or maybe **→** go to Q4
- No **→** exclude and record reason

1. Does the publication address environmental justice, inequity, or disparity—explicitly or implicitly?

- Yes or maybe **→** go to Q5
- No **→** exclude and record reason

1. Is the publication a primary research study (qualitative, quantitative or mixed methods) or grey literature from a reputable organization?

- Yes or maybe **→** Include for full-text screening
- No **→** exclude and record reason

**Stage 2 Full-text Screening Form**

Instructions: Answer sequentially. If the answer is NO, exclude and record the reason in Excel.

1. Is the study conducted in or focused on the Global South, including Africa, Latin America, Asia or the Middle East?

- Yes **→** go to Q2
- No **→** exclude and record reason

1. Does the study describe human exposure to pesticides through occupational, environmental, community, or domestic pathways? (e.g. handling, pesticide drift, reuse of containers, poisonings etc.)?

- Yes **→** go to Q3
- No **→** exclude and record reason

1. Is the study on a population affected by pesticide exposure (e.g. farmers, workers, vendors, children, migrants etc.)?

- Yes **→** go to Q4
- No **→** exclude and record reason

1. Does the study apply a justice lens—distributive, procedural, recognition, capabilities, epistemic—or discuss pesticide-related inequity, disparity, or rights-based issues?

- Yes **→** go to Q5
- No **→** exclude and record reason

1. Does the study present primary data (qualitative, quantitative or mixed methods) or grey literature from a credible source (e.g. UN, WHO, FAO, PAN, IPEN etc.)

- Yes **→** Include for review
- No **→** exclude and record reason

# Table S3. Preferred Reporting Items for Systematic reviews and Meta-Analyses extension for Scoping Reviews (PRISMA-ScR) Checklist

| **Section** | **Item** | **PRISMA-ScR Checklist Item** | **Reported on Page #** |
| --- | --- | --- | --- |
| **TITLE** | | | |
| Title | 1 | Identify and report as a scoping review. | 1 |
| **ABSTRACT** | | | |
| Structured summary | 2 | Provide a structured summary that includes (as applicable): background, objectives, eligibility criteria, sources of evidence, charting methods, results, and conclusions that relate to the review questions and objectives. | 1-2 |
| **INTRODUCTION** | | | |
| Rationale | 3 | Describe the rationale for the review in the context of what is already known. Explain why the review questions/objectives lend themselves to a scoping review approach. | 2-3 |
| Objectives | 4 | Provide an explicit statement of the questions and objectives being addressed with reference to their key elements (e.g., population or participants, concepts, and context) or other relevant key elements used to conceptualize the review questions and/or objectives. | 3 |
| **METHODS** | | | |
| Protocol and registration | 5 | Indicate whether a review protocol exists; state if and where it can be accessed (e.g., a Web address); and if available, provide registration information, including the registration number. | 3 |
| Eligibility criteria | 6 | Specify characteristics of the sources of evidence used as eligibility criteria (e.g., years considered, language, and publication status), and provide a rationale. | 4-5 |
| Information sources | 7 | Describe all information sources in the search (e.g., databases with dates of coverage and contact with authors to identify additional sources), as well as the date the most recent search was executed. | 4 |
| Search | 8 | Present the full electronic search strategy for at least 1 database, including any limits used, such that it could be repeated | Supplemental material Table S2 |
| Selection of sources of evidence | 9 | State the process for selecting sources of evidence (i.e., screening and eligibility) included in the scoping review | 5-6 |
| Data charting process | 10 | Describe the methods of charting data from the included sources of evidence (e.g., calibrated forms or forms that have been tested by the team before their use, and whether data charting was done independently or in duplicate) and any processes for obtaining and confirming data from investigators. | 6-7 |
| Data items | 11 | List and define all variables for which data were sought and any assumptions and simplifications made. | 6 |
| Critical appraisal of individual sources of evidence | 12 | If done, provide a rationale for conducting a critical appraisal of included sources of evidence; describe the methods used and how this information was used in any data synthesis (if appropriate). | N/A |
| Synthesis of results | 13 | Describe the methods of handling and summarizing the data that were charted. | 6 |
| **RESULTS** | | | |
| Selection of sources of evidence | 14 | Give numbers of sources of evidence screened, assessed for eligibility, and included in the review, with reasons for exclusions at each stage, ideally using a flow diagram. | 6-7 |
| Characteristics of sources of evidence | 15 | For each source of evidence, present characteristics for which data were charted and provide the citations. | Table S3 |
| Critical appraisal within sources of evidence | 16 | If done, present data on critical appraisal of included sources of evidence (see item 12). | N/A |
| Results of individual sources of evidence | 17 | For each included source of evidence, present the relevant data that were charted that relate to the review questions and objectives. | 7-20 |
| Synthesis of results | 18 | Summarize and/or present the charting results as they relate to the review questions and objectives | 7-20 |
| **DISCUSSION** | | | |
| Summary of evidence | 19 | Summarize the main results (including an overview of concepts, themes, and types of evidence available), link to the review questions and objectives, and consider the relevance to key groups. | 20-24 |
| Limitations | 20 | Discuss the limitations of the scoping review process | 24 |
| Conclusions | 21 | Provide a general interpretation of the results with respect to the review questions and objectives, as well as potential implications and/or next steps | 24-25 |
| **FUNDING** | | | |
| Funding | 22 | Describe sources of funding for the included sources of evidence, as well as sources of funding for the scoping review. Describe the role of the funders of the scoping review. | 25 |

# Table S4. List of Included Studies

1. Acero, C., et al., *Navigating Chemical Toxicity in Coca Production in the Colombian Borderlands of Putumayo.* Medical Anthropology, 2023. **42**(7): p. 650-666.
2. Andersson, E. and E. Isgren, *Gambling in the garden: Pesticide use and risk exposure in Ugandan smallholder farming.* Journal of Rural Studies, 2021. **82**: p. 76-86.
3. Arancibia, F. and R. Motta, *Undone Science and Counter-Expertise: Fighting for Justice in an Argentine Community Contaminated by Pesticides.* Science As Culture, 2019. **28**(3): p. 277-302.
4. Balayannis, A., *Toxic sights: The spectacle of hazardous waste removal.* Environment And Planning D-Society & Space, 2020. **38**(4): p. 772-790.
5. Breilh, J., N. Pagliccia, and A. Yassi, *Chronic pesticide poisoning from persistent low-dose exposures in Ecuadorean floriculture workers: toward validating a low-cost test battery.* Int J Occup Environ Health, 2012. **18**(1): p. 7-21.
6. Brisbois, B., *Bananas, pesticides and health in southwestern Ecuador: A scalar narrative approach to targeting public health responses.* Soc Sci Med, 2016. **150**: p. 184-91.
7. Brisbois, B.W., J.M. Spiegel, and L. Harris, *Health, environment and colonial legacies: Situating the science of pesticides, bananas and bodies in Ecuador.* Soc Sci Med, 2019. **239**: p. 112529.
8. Christie, M.E., E. Van Houweling, and L. Zseleczky, *Mapping gendered pest management knowledge, practices, and pesticide exposure pathways in Ghana and Mali.* Agriculture And Human Values, 2015. **32**(4): p. 761-775.
9. Cole, D.C., et al., *Community and household socioeconomic factors associated with pesticide-using, small farm household members' health: a multi-level, longitudinal analysis.* Int J Equity Health, 2011. **10**: p. 54
10. Corriols, M. and A. Aragorn, *Child labor and acute pesticide poisoning in Nicaragua: failure to comply with children's rights.* Int J Occup Environ Health, 2010.
11. Creed, I.F., et al., *A geo-gender-based analysis of human health: The presence of cut flower farms can attenuate pesticide exposure in African communities, with women being the most vulnerable.* J Glob Health, 2024. **14**: p. 04064.
12. de Souza, R.S., I.B. Barbieri, and M.Z. Adriano, *Agrochemical contamination in Brazil seen as a crime of ecocide. Towards an ecocentric approach on pesticide regulation.* Desenvolvimento E Meio Ambiente, 2021. **57**: p. 229-244.
13. Dinham, B. and S. Malik, *Pesticides and Human Rights.* International Journal of Occupational and Environmental Health, 2003. **91**(1): p. 40-52.
14. Frey, R.S., *The international traffic in pesticides.* Technological Forecasting and Social Change, 1995. **50**(2): p. 151-169.
15. Frey, R.S., *Agent Orange and America at war in Vietnam and Southeast Asia.* Human Ecology Review, 2013. **20**(1): p. 1-10.
16. Galt, R.E., *Beyond the circle of poison: Significant shifts in the global pesticide complex, 1976–2008.* Global Environmental Change, 2008. **18**: p. 786-799.
17. Gamlin, J., *Huichol Migrant Laborers and Pesticides: Structural Violence and Cultural Confounders.* Med Anthropol Q, 2016. **30**(3): p. 303-20.
18. Hurtado, D. and I. Vélez-Torres, *Toxic Dispossession: On the Social Impacts of the Aerial Use of Glyphosate by the Sugarcane Agroindustry in Colombia.* Critical Criminology, 2020. **28**(4): p. 557-576.
19. Human Rights Watch, *Ripe with abuse: Human rights conditions in South Africa's fruit and wine industries*. 2011, Human Rights Watch: New York. p. 11
20. Isgren, E. and E. Andersson, *An Environmental Justice Perspective on Smallholder Pesticide Use in Sub-Saharan Africa.* Journal Of Environment & Development, 2021. **30**(1): p. 68-97.
21. Krishna, A., *Tales Behind a Spice: Toxified Terrain and Tortured Bodies in the Making of Indian Small Cardamom.* Global Environment, 2024. **17**(2): p. 281-310
22. Lapegna, P. and J. Kunin, *Ambiguities at Sites of Acceptance: Agrarian Neoliberalism and Herbicide Exposure in Argentina.* Environmental Justice, 2023. **16**(1): p. 82-88.
23. London, L. and H.A. Rother, *People, pesticides, and the environment: who bears the brunt of backward policy in South Africa?* New Solut, 2000. **10**(4): p. 339-50.
24. London, L., *Human rights, environmental justice, and the health of farm workers in South Africa.* Int J Occup Environ Health, 2003. **9**(1): p. 59-68.
25. Lundsteen, S., *Shadow Places, Environmental Justice, and the Submergence of Pollution.* Environmental History, 2024. **29**(2): p. 281-306.
26. Lyons, K., *Chemical warfare in Colombia, evidentiary ecologies and ‘senti-actuando’ practices of justice.* Social Studies of Science, 2018. **48**(3): p. 414-437.
27. Melangadi, F., *Environmental Crime and Victimization: A Green Criminological Analysis of the Endosulfan Disaster, Kasargod, Kerala.* International Annals of Criminology, 2017. **55**(2): p. 216-230.
28. Mendez, A., et al., *Tracking pesticide fate in conventional banana cultivation in Costa Rica: A disconnect between protecting ecosystems and consumer health.* Sci Total Environ, 2018. **613-614**: p. 1250-1262.
29. Moraes, N.G.D., et al., *Ethnic-racial disparities in poisoning cases: analysis of drugs of abuse, medicines and pesticides in Brazil.* Journal Of Toxicology and Environmental Health, 2024. **87**(21): p. 863-878.
30. Noory, B., R.R. Habib, and I. Nuwayhid, *Exposure of Syrian refugee agricultural workers to pesticides in Lebanon: a socio-economic and political lens.* Front Public Health, 2024. **12**: p. 1402511.
31. Orozco, F.A., et al., *Monitoring adherence to the international code of conduct: highly hazardous pesticides in central Andean agriculture and farmers' rights to health.* Int J Occup Environ Health, 2009. **15**(3): p. 255-68.
32. Pauker, S., *"Spraying first and asking questions later": Congressional efforts to mitigate the harmful environmental, health, and economic impacts of US-sponsored coca fumigation in Colombia.* ECOLOGY LAW QUARTERLY, 2003. **30**(3): p. 661-692.
33. Rosenthal, E., *The tragedy of Tauccamarca: a human rights perspective on the pesticide poisoning deaths of 4 children in the Peruvian Andes.* Int J Occup Environ Health, 2003. **9**(1): p. 53-8.
34. Rother, H.A., *Falling through the regulatory cracks: Street selling of pesticides and poisoning among urban youth in South Africa.* Int J Occup Environ Health, 2010. **16**(2): p. 202-13.
35. Rother, H.A., *Pesticide Vendors in the Informal Sector: Trading Health for Income.* New Solutions, 2016. **26**: p. 241-252.
36. Sass, R., *Agricultural 'killing fields': The poisoning of Costa Rican banana workers.* International Journal of Health Services, 2000. **30**(3): p. 491-514.
37. Satheesh, S., *Development as recolonization: the political ecology of the Endosulphan disaster in Kasargod, India.* Critical Asian Studies, 2017. **49**(4): p. 587-596.
38. Shattuck, A., *Toxic Uncertainties and Epistemic Emergence: Understanding Pesticides and Health in Lao PDR.* Annals of the American Association of Geographers, 2021. **111**(1): p. 216-230.
39. Shattuck, A., *Risky subjects: Embodiment and partial knowledges in the safe use of pesticide.* Geoforum, 2021. **123**: p. 153-161.
40. Sony, R.K., D. Münster, and S. Krishnana, *What counts as evidence? Examining the controversy over pesticide exposure and etiology in an environmental justice movement in Kerala, India..* Environmental Sociology, 2022, **9**: p.148.
41. Swartz, A., et al., *Toxic layering through three disciplinary lenses: childhood poisoning and street pesticide use in Cape Town, South Africa.* Med Humanit, 2018. **44**(4): p. 247-252.
42. Terwindt, C., S. Morrison, and C. Schliemann, *Health rights impacts by agrochemical business: Legally challenging the “myth of safe use".* Utrecht Journal of International and European Law, 2018. **34**(2): p. 130-145.
43. United Nations Commission on Human Rights (UNCHR), *Report by Special Rapporteur (02 August 2016) UN Doc A/HRC/39/48.* 2016.
44. UNCHR, *Report by Special Rapporteur (03 August 2018) UN Doc A/HRC/39/48.* 2018.
45. UNCHR, *Report by Special Rapporteur (03 August 2018) UN Doc A/HRC/39/48/Add.1.* 2018.
46. UNCHR, *Report by Special Rapporteur B Tuncak (07 October 2019) A/74/480.* 2019.
47. UNCHR, *Report by Special Rapporteur B Tuncak (05 August 2020) UN Doc A/75/290.* 2020.
48. UNCHR, *Report by Special Rapporteur B Tuncak (05 August 2021) UN Doc A/HRC/45/12/Add.2.* 2021.
49. UNCHR, *Report by Special Rapporteur M Orellana (28 July 2022)UN Doc A/77/183.* 2022.
50. UNCHR, *Report by Special Rapporteur M Orellana (14 July 2023) UN Doc A/HRC/54/25/Add.1.* 2023.
51. UNCHR, *Report by Special Rapporteur M Orellana (16 July 2024) UN Doc A/79/163.* 2024.
52. UNCHR, *Report by Special Rapporteur M Orellana (17 July 2024) UN Doc A/HRC/57/52/Add.1.* 2024.
53. Utyasheva, L., et al., *Stop blaming the farmer: Dispelling the myths of 'misuse' and 'safe' use of pesticides to protect health and human rights.* Journal Of Human Rights, 2024. **23**(3): p. 231-252.
54. Varona, E.M., et al., *Organochlorine pesticide exposure among agricultural workers in Colombian regions with illegal crops: an exploration in a hidden and dangerous world.* International Journal of Environmental Health Research, 2010. **20**(6): p. 407-414.

# Table S5: Characteristics of included studies

| Reference | Study location | Study design | Study population (sample size) | Gender distribution (percentage)^[[1]](#footnote-1)^ | Age range (percentage of participants) | Data collection methods | Data analysis | Key findings |
| --- | --- | --- | --- | --- | --- | --- | --- | --- |
| Acero et al. 2023 | Putumayo, Colombia | Qualitative | Coca farmers (n=8) | Male = 50%  Female = 50% | Not provided | Participant observations  Semi-structured interviews | Narrative and Thematic analysis | Coca farmers experience pesticide exposure due to structural inequalities, including uneven rural development, criminalization, and lack of access to health and legal protections. |
| Andersson and Isgren 2021 | Torroro, Uganda | Mixed methods | Smallholder farmers (n=2000) | Male = 68%  Female = 32% | <29 years = 14.5%  30-45 years = 45%  50 years= 40.5% | Household surveys  In-depth interviews  Focus group discussions  Field observations | Descriptive statistics  Thematic analysis | Smallholder farmers in Uganda face disproportionate pesticide exposure due to systemic regulatory failures, economic barriers, and gendered inequities. |
| Arancibia and Motta 2019 | Ituzaingó Anexo, Argentina | Qualitative | Environmental justice movement members (n=9, NGO members (n=3), doctors (n=3), scientists (n=3) | Not provided | Not provided | Interviews | Narrative and Thematic analysis | Rural communities adjacent to genetically modified soybean fields leveraged social mobilization, scientific and legal expertise to challenge pesticide exposure and achieve procedural justice in criminal convictions for illegal aerial spraying of glyphosate. |
| Balayannis 2020 | Vikuge, Tanzania | Ethnography | Stakeholders involved in pesticide stockpile removal (number not specified) | Not provided | Not provided | Participant observation  Archival research  Interviews | Narrative and Thematic analysis | Expired pesticide stockpile in Tanzania reinforced colonial environmental injustices by prioritizing visual remediation over substantive decontamination. |
| Breilh et al. 2012 | Granobles River Basin, Cayambe, Ecuador | Cross-sectional and clinical tests | Agricultural workers aged between 18 to 69 years (n=123). | Male = 64.2% Female = 35.8% | Average age = 32.6 years (SD=9.7). | Questionaires (Pentox and EpiStress)  Clinical blood test for acetylcholinesterase (AChE) | Descriptive statistics  Factor analysis  Logistic Regression  Canonical discriminant analysis | Floriculture workers face chronic pesticide poisoning risks due to inadequate occupational health protections and weak regulatory enforcement. |
| Brisbois 2016 | El Oro Province, Ecuador | Ethnography | Farmers, plantation workers, and community members exposed to pesticides in banana production (n=30) | Male = 77%  Female = 23% | 40-70 years | Participant observations  Key informant interviews  Semi-structured interviews | Narrative and Thematic analysis | Structural economic inequalities and unsafe labor conditions contribute to pesticide exposure among banana plantation workers |
| Brisbois et al. 2019 | Coastal regions of Ecuador | Ethnography | Farmers, plantation workers, and community members exposed to pesticides in banana production | Not provided | Not provided | Secondary sources | Narrative and Thematic analysis | Corporate-driven toxicological science and postcolonial inequalities marginalize vulnerable populations' knowledge and health risks from pesticides. |
| Christie et al. 2015 | Tuobadom, Brong Ahafo Region, Ghana.  Ouelesseboug-ou, Mali. | Mixed methods | Smallholder tomato farmers in Ghana (n=283) and Mali (n=120) | Ghana:  Male = 74%  Female = 26%  Mali:  Male = 50%  Female = 50% | Not provided | Household surveys  Focus Group Discussions  Semi-structured interviews  Participatory mapping  Participant observation | Descriptive statistics  Thematic analysis | The gender division of labor and differences in access to resources, information, and power lead to gendered pesticide exposure pathways |
| Cole et al. 2011 | Central and Northen Ecuador | Longitudinal | Potato smallholder farmer (manager) (n=380) or household manager (n=376) between 18-65 years, lived in the community during the past three years. | Crop manager: Male = 87% Female = 13%  Household manager:  Male = 3%  Female = 97% | Household members: average age 39.8 years (SD=12.3).  Crop managers: average age = 41.8 years (SD=13.1). | Household surveys  Neurological assessment | Descriptive statistics  Multiple regression | Participatory training improved pesticide safety knowledge among Ecuadoran farmers, but broader systemic injustices remained unaddressed. |
| Corriols and Aragorn 2010 | Nicaragua | Retrospective observational | Acute pesticide poisoning (APP) cases involving children aged 5–14 years engaged in agricultural work between 1995-2006 (n=432), of which 6 cases resulted in death. | APP cases:  Boys= 77%  Girls = 23%  Mortality:  Boys= 67%  Girls= 33% | Average age of APP cases= 13.6 years, and most cases between 10-14 years.  Mortality: most cases between 12-14 years | Poison surveillance data | Descriptive statistics | National pesticide bans in Nicaragua reduced poisoning incidents of child labourers but systemic rural poverty continues to drive hazardous child labour. |
| Creed et al. 2014 | Naivasha and Mogotio, Kenya | Case-control | Residents of Naivasha (industrial cut flower hub) and Mogotio (rural agricultural community).    Naivasha socieconomic survey (n=800) and biological samples (n=64).    Mogotio socio-economic survey (n=200) and biological samples (n=25) | Naivasha:  Male = 37%  Female = 63%  Mogotio:  Male = 37% Females = 69%  Biological samples (n=89):  Male = 34%  Female = 66% | Naivasha: 56% of the population below 30 years | Socio-economic survey  Biological samples | Descriptive statistics  Inferential statistics  Random Forest Analysis | There was lower awareness and risk mitigation in non-industrial agricultural areas and that regulatory measures and risk awareness campaigns helped reduce occupational exposure but does not fully protect surrounding communities. |
| de Souza et al. 2021 | Brazil | Policy analysis | General population affected by pesticide exposure, with a focus on vulnerable communities in Brazil | Not provided | Not provided | Documents | Textual analysis  Narrative and thematic analysis | Brazil’s pesticide regulations institutionalize environmental injustice by enabling the use of banned substances and exposing vulnerable populations to toxic risks. |
| Dinham and Malik 2003 | Less developed countries (LDCs) | Policy analysis | agricultural workers, small-scale farmers, and rural communities in countries where pesticide regulations are weak | Not provided | Not provided | International human rights instrument documents  Legal cases | Thematic analysis | Marginalized rural communities, women, and children disproportionately bear the health burdens of pesticide exposure under weak global regulatory systems. |
| Frey 1995 | Less developed countries | Policy analysis | agricultural workers and communities | Not provided | Not provided | Documents | Thematic analysis | Pesticide harm in less developed countires reflects global economic dependencies that externalize environmental and health risks onto poor rural workers. |
| Frey 2013 | Vietnam  Laos  Cambodia | Policy analysis | Rural communities in Vietnam, Laos and Cambodia | Not provided | Not provided | Documents | Thematic analysis | The U.S. military's use of herbicides like Agent Orange caused widespread environmental damage and severe health consequences which exemplifies environmental injustice by transferring risks to marginalized communities. |
| Galt 2008 | Northen Cartago and Ujarras Valley, Costa Rica | Case study | Farmers in Costa Rica involved in export and domestic agricultural production | Not provided | Not provided | Documents | Thematic analysis | Developing countries have transitioned from banned organochlorines to newer classes of pesticides, often more acutely toxi with farmworkers in developing countries face the greatest exposure risks due to shifts in pesticide classes. |
| Gamlin 2016 | Nayarit State and highlands, Mexico | Ethnography | Huichol indigenous migrant laborers, including men, women, and children, who work on tobacco plantations. | Not provided | Not provided | Semi-structured interviews  Participant observation | Narrative and thematic analysis | Indigenous and rural women in Mexico suffer compounded pesticide exposures due to gendered labor dynamics and systemic neglect of their health rights. |
| Hurtado and Velez-Torres 2020 | Cauca River Valley, Colombia | Qualitative | Afro-descendant and Indigenous communities living near sugarcane monocultures who have lived in the area for at least twenty years and engage in traditional agricultural activities (n=15) | Not provided | Not provided | Semi-structured interviews | Thematic analysis | Aerial spraying of glyphosate undermines Afro-descendant and Indigenous communities' rights to health, food, and water. |
| Human Rights Watch 2011 | Western Cape, South Africa | Report | Former and current farmworkers (n= 117) and farm dwellers (n=16) | Not provided | Not provided | Interviews | Thematic analysis | Aerial spraying of glyphosate has caused severe socio-environmental harm in rural communtiies |
| Isgren and Andersson 2021 | Uganda | Case Study | Rural smallholder farmers | Not provided | -Not provided | Documents | Thematic analysis | Weak pesticide governance in Sub-Saharan Africa exacerbates smallholder exposure, highlighting the inadequacy of "safe use" approaches and need for structural reforms. |
| Krishna 2024 | Cardamon Hills, Kerala, India | Ethnography | Lower-caste women workers in cardamom plantations | All women | Not provided | Participant observation  Semi-structured interviews | Narrative and thematic analysis | Caste and gender-based labor hierarchies in cardamom plantations expose marginalized women workers to disproportionate pesticide harms. |
| Lapegna and Kunin 2023 | La Pampa, Buenos Aires, Cordoba, Argentina | Qualitative | Farmers, agronomists, rural journalists, farm insurance agents (n=14) | Not provided | Not provided | In-depth interviews | Thematic analysis | Farmers experience contradictory pressures to embrace herbicide use while suffering health and environmental degradation. |
| London 2003 | South Africa | Case study | Fruit farm workers | Not provided | Not provided | Documents | Thematic analysis | Historical systemic inequalities rooted in apartheid continue to expose South African farm workers to hazardous pesticides with limited labor protections. |
| London and Rother 2000 | South Africa | Policy analysis | Farm workers, small-scale farmers, and rural residents | Not provided | Not provided | Documents | Thematic analysis | Pesticide policies in South Africa disproportionately harm vulnerable farm workers and smallholders, reflecting fragmented governance and exclusion from decision-making. |
| Lundsteen 2024 | Gujarat, India | Case study | Agricultural workers | Not provided | Not provided | Documents | Thematic analysis | Corporate displacement of pollution from Denmark to India reveals how global pesticide production perpetuates environmental injustice and colonial exploitation. |
| Lyons 2018 | Putumayo, Colombia | Ethnography | Farmer compensation claimants affected by aerial spraying (n=71) | Not provided | Not provided | Participant observation  Interviews  Documents | Narrative and thematic analysis | War-on-drugs policies in Colombia criminalized rural communities and exacerbated environmental injustices through aerial pesticide fumigation. |
| Melangadi 2017 | Kasargod, Kerala, India | Case study | Rural communities living near cashew plantations | Not provided | Not provided | Documents | Thematic analysis | Prolonged endosulfan spraying in India caused systemic health and environmental harm, exemplifying the failure of legal and regulatory systems to deliver environmental justice |
| Mendez et al. 2017 | Caño Azul River drainage area, Costa Rica | Human exposure modelling | Agricultural workers and rural communities near banana plantations | Not provided | Not provided | Chemical transport modelling | Ecotoxicology modelling  Uncertainty and sensitivity analysis | Banana-exporting regions in the country experience local environmental degradation and health risks while regulatory systems prioritize consumer safety abroad. |
| Moraes et al, 2024 | Brazil | Ecological | Pesticide poisoning cases from the national health database (n=41254) | Male=56%  Female= 44% | <4 years old = 2.3%.  10-19 years = 12.3%.  20-59 years= 67.8% .  > 60 years= 6.5% | Poisoning notifications | Descriptive statistics  Correlation | Non-white and low-income populations in Brazil experience disproportionately higher poisoning rates from pesticides, revealing structural racism in public health. |
| Noory et al., 2024 | Lebanon | Case study | Syrian refugee agricultural workers | Not provided | Not provided- | Literature, policy and legal documents | Thematic analysis | Syrian refugee workers in Lebanon face extreme pesticide exposure driven by systemic labor exploitation, deregulation, and structural exclusion. |
| Orozco et al. 2009 | Ecuador and Peru | Mixed methods | Surveys farmers Peru (n=714) and Ecuador (n=480). Inclusion criteria- Smallholder farmers and farmworkers engaged in potato and grain cultivation between 18-65 years, lived in community past 3 years, and literate in Spanish. | Not provided | Not provided | Household surveys  Focus Group Discussions Field observations | Descriptive statistics and thematic analysis | Smallholder farmers suffer from pesticide exposure and unsafe disposal practices due to systemic regulatory failures and language barriers. |
| Pauker 2003 | Colombia | Legal analysis | Rural Colombian communities, including indigenous groups and small-scale farmers | Not provided | - Not provided | Documents | Thematic analysis | Aerial fumigation campaigns in Colombia displaced rural communities, undermining food security and indigenous and rural populations to environmental harm. |
| Rosenthal 2003 | Cajarmaca Peru | Case study | Quechua-speaking Indigenous community in the Peruvian Andes | Not provided | Not provided | Documents | Thematic analysis | Regulatory and corporate negligence led to a mass pesticide poisoning of Indigenous children in Peru. |
| Rother 2010 | Cape Town, South Africa | Quali | Youth engaged in selling, transporting, and handling pesticides. | Not provided | Not provided | Interviews  Field observations | Thematic analysis | Informal sales of toxic pesticides in South Africa expose vulnerable urban populations to uncontrolled health hazards. |
| Rother 2016 | Cape Town, South Africa | Qualitative | Informal pesticide vendors | Not provided | Vendors include children, women, and unemployed youth. | Field observations  In-depth interviews | Thematic analysis | Street pesticides sold in unregulated markets cause severe health risks, revealing systemic governance failures. |
| Sass 2000 | Costa Rica | Policy analysis | Banana plantation workers | Not provided | Not provided | Documents | Thematic analysis | Widespread use of DBCP on banana plantations caused mass sterilization and chronic illnesses among exposed workers. |
| Satheesh 2017 | Kasaragod, Kerala, India | Ethnography | Endosulfan victims/ survivors, residents living near plantations, and local environmental activists (n=50) | Not provided | Not provided | In-depth interviews | Narrative and thematic analysis | The Endosulfan disaster in India reflects how systemic injustice delayed recognition and reparation for pesticide victims. |
| Shattuck 2019 | Laos | Mixed methods | professional pest applicators (n=27), Maize farmers that recently transitioned from subsistence farming to commercial monoculture agriculture (n=86), NGO personnel (n=31) | Not provided | Not provided | Household surveys  Semi-structured interviews  Participant observation | Descriptive statistics  Thematic analysis | The "safe use" model for pesticide exposure is fundamentally flawed because it blames farmers instead of addressing the structural constraints, such as economic precarity that forces farmers to accept pesticide risks. |
| Shattuck 2021 | Laos | Ethnography | smallholder farmers who use pesticides in agricultural production | Not provided | Not provided | Field observations | Thematic analysis | Pesticide risks in smallholder agriculture are poorly understood and inadequately addressed, reinforcing environmental injustice. |
| Sony et al. 2023 | Kasaragod, Kerala, India | Qualitative | environmental activists (n=6), agricultural scientists (n=2), NGO personnel (n=6), advocates (n=3) | Not provided | Not provided | Interviews  Documents | Thematic analysis | Government and corporate actors exploited scientific uncertainty to delay justice for communities exposed to endosulfan in Kerala, India. |
| Swartz et al., 2018 | Cape Town, South Africa | Ethnography | Children poisoned by street pesticides (n=4) admitted to Red Cross War Memorial Children's Hospital | Not provided | Age ranging from 8 months to 12 years | Participant observation | Narrative and thematic analysis | Children in urban informal settlements are poisoned by illegal pesticides due to systemic failures in housing, pest control, and poverty alleviation policies. |
| Terwindt et al., 2018 | Low and middle income countries (LMICs) | Legal analysis | Farmers, plantation workers, and communities exposed to highly hazardous pesticides. | Not provided | Not provided | Documents- legal cases, international regulations and corporate strategies | Textual analysis  Thematic analysis | Structural barriers in international law and corporate practices prevent farmers in the Global South from achieving justice after pesticide exposure. |
| United Nations Commission on Human Rights (UNCHR 2016) | LMICs | Report | Children | Not provided | Not provided |  |  | Children working in agriculture continue to use hazardous pesticides  despite the bans on such products in several countries, raising questions of double standards and discrimination |
| UNCHR 2018a | LMICs | Report | Agricultural workers and child labourers | Not provided | Not provided |  |  | The illegal use of banned pesticides and toxic chemicals, as well as of counterfeit products, standards of continues to be a major problem globally, a serious threat to children of the workers affected, to  protection communities and to consumers |
| UNCHR 2018b | Sierra Leone | Report | Agricultural workers | Not provided | Not provided |  |  | The Special Rapporteur points out that, in some situations, the Government seems to have adopted contradictory policies to stimulate the purchase of toxic products in order to boost economic activity, despite limited resources and capacity for their management. Most victims of toxics have no access to justice, nor are they aware of effective remedies, and most perpetrators of violations relating to toxics are not held accountable. The burden of proving the cause of illness, the lack of information, the insurmountable costs of judicial remedy, corporate structures, global and devolved supply chains and other factors obstruct the path to justice and remedy for most victims (ibid., para. 18). The Special Rapporteur notes in particular that in Sierra Leone no cases dealing specifically with pollution, contamination and exposure to hazardous substances and wastes have been brought by victims to the courts. |
| UNCHR 2019 | LMICs | Report |  |  |  |  |  | documents systemic failures to protect human rights from pesticide harms in low- and middle-income countries. |
| UNCHR 2020 | LMICs | Report | Human rights activists, agricultural workers, child labourers | Not provided | Not provided |  |  | High-income States continue to export highly hazardous pesticides and toxic industrial chemicals, resulting in widespread infringements in low and middle-income countries of the human rights to life, dignity and freedom. In 2019 , at least 30 States exported hazardous substances that had been banned locally for health and environmental reasons to Latin America, Africa and Asia. This practice was denounced by 36 United Nations experts, who called for it to end |
| UNCHR 2021 | Brazil | Report | Landless workers, communities exposed to aerial spraying, indigenous communities, human rights activists | Not provided | Not provided |  |  | There is a consistent pattern of not providing advance notice of spraying or information about pesticides used. For example, landless workers decried that the only “notice” they had received was a cloud of chemicals above their encampments. The underreporting of pesticide poisoning is a major concern, and it is estimated that, for each case for which notification is received, 50 remain unreported. |
| UNCHR 2022 | LMICs | Report | Indigenous communities | Not provided | Not provided |  |  | Access to justice for indigenous peoples for the adverse effects of toxics on their lands and health is limited and often illusory. Minimal financial resources, State discrimination and corruption, and a lack of protective laws cement the continued marginalization of indigenous people. |
| UNCHR 2023 | Ghana | Report | Agricultural workers | Not provided | Not provided |  |  | Raisesed concern that some of the agrochemicals used in Ghana are banned for use in Europe and elsewhere because they are hazardous to human health and the environment. Examples include the herbicides atrazine and paraquat and the pesticide chlorpyrifos.48 It is also alarming that one of the most widely used herbicides in the country is glyphosate (locally known as kondem), which the International Agency for Research on Cancer has classified as probably carcinogenic to humans. Even though sustainable farming practices are available, farmers have little knowledge of them, and the Government mainly promotes pesticides. |
| UNCHR 2024a | LMICs | Report | Famers and farmworkers and their families | Not provided | Not provided |  |  | Pesticides and chemical-based farming dramatically undermine women’s power regarding their roles as food producers, caretakers of biodiversity and seed keepers. Furthermore, in many countries the use of costly pesticides and commercial seeds has augmented women’s poverty.. |
| UNCHR 2024b | South Africa | Report | Famers and farmworkers and their families, communities affected by pesticide-related disasters, victims of domestic and and street pesticide use, including children | Not provided | Not provided |  |  | The Special Rapporteur also encourages South Africa to address outstanding gaps. Among these, public participation and mechanisms for the traceability of pesticides are needed. Additionally, pesticide labelling requirements should be in languages accessible to workers and include clear specifications on buffer zones, non-target areas and aerial spraying.. |
| Utyasheva et al., 2024 | LMICs | Policy analysis | Small-holder farmers, farm workers, and agricultural communities in LMICs | Not provided | Not provided | Literature and legal and policy documents | Thematic analysis | Blaming farmers for "misuse" of pesticides masks structural causes of hazardous exposures rooted in weak governance. |
| Varona et al., 2010 | Colombia | Cross-sectional | agricultural workers in an area growing illegal crops and had used pesticides in farming activities during the past two years (n=112) | Not provided | Not provided | Biological samples | Descriptive statistics | Banned organochlorine pesticides persist in agricultural communities, highlighting failures in international pesticide governance. |

^1^Sex as reported in original studies. M = male; F = female. If not specified, sex was assumed based on language used (e.g., male/female).

Improved pesticide safety practices among farmers and increased gender-sensitive knowledge [58, 59].

1. As reported in original studies. If not specified, it was assumed based on language used (e.g., male/female or boys/girls). [↑](#footnote-ref-1)
